# Modulating Temporal and Spatial Oxygenation over Adherent Cellular Cultures

**DOI:** 10.1371/journal.pone.0006891

**Published:** 2009-09-03

**Authors:** Shawn C. Oppegard, Ki-Hwan Nam, Janai R. Carr, Stacey C. Skaalure, David T. Eddington

**Affiliations:** 1 Department of Bioengineering, University of Illinois at Chicago, Chicago, Illinois, United States of America; 2 Department of Biochemistry and Molecular Genetics, University of Illinois at Chicago, Chicago, Illinois, United States of America; Texas A&M University, United States of America

## Abstract

Oxygen is a key modulator of many cellular pathways, but current devices permitting *in vitro* oxygen modulation fail to meet the needs of biomedical research. A microfabricated insert for multiwell plates has been developed to more effectively control the temporal and spatial oxygen concentration to better model physiological phenomena found *in vivo*. The platform consists of a polydimethylsiloxane insert that nests into a standard multiwell plate and serves as a passive microfluidic gas network with a gas-permeable membrane aimed to modulate oxygen delivery to adherent cells. Equilibration time is on the order of minutes and a wide variety of oxygen profiles can be attained based on the device design, such as the cyclic profile achieved in this study, and even oxygen gradients to mimic those found *in vivo*. The proper biological consequences of the device's oxygen delivery were confirmed in cellular models via a proliferation assay and western analysis of the upregulation of hypoxia inducible transcription factor-1α. These experiments serve as a demonstration for the platform as a viable tool to increase experimental throughput and permit novel experimental possibilities in any biomedical research lab.

## Introduction

Oxygen is a key metabolic variable influencing many different biological phenomena; however, current tools to probe this variable are either crude, inefficient, or have not changed since the dawn of cell culture techniques. Oxygen is increasingly implicated in many signaling pathways and one route of modulation is through the hypoxia-inducible factor (HIF) family of heterodimeric transcription factors, which regulate the cellular response to oxygen tension [1]. HIF-1α is a ubiquitous transcription factor that has been found to influence development, glucose metabolism, apoptosis, solid tumor growth, and angiogenesis, among many other functions [2]. Enabling a simple tool to explore these cellular behaviors would greatly facilitate these investigations and accelerate scientific discovery.

Tissues are often exposed to complex oxygen concentrations spatially and temporally *in vivo*, which addresses the requirement for a device that has defined control over the oxygen environment exposed to cells *in vitro*. Gradients of oxygen are standard byproducts of cellular metabolism found throughout every tissue and across all organisms. These gradients of oxygen are increasingly highlighted as crucial metabolic regulators when studying drug toxicity [3], [4], the hematopoietic stem cell niche [5], plant biology [6], [7], liver zonation [8], [9], and throughout developmental biology [10], [11]. Intermittent hypoxia, or cycling oxygenation, has been shown to be important in a number of pathological conditions, such as in sleep apnea [12] and tumor development [13]. These studies have demonstrated that cells do indeed respond differently to variable oxygen levels as opposed to constant oxygenation, and often times the former is more pathological. Thus, replicating both these *in vivo* gradients and transient oxygenation in an *in vitro* model accessible to any standard cell biology lab would have a huge impact across many fields.

The hypoxic chamber remains as the tool of choice and has seen a great deal of use in applications requiring control over the oxygen tension exposed to cells due to its ease of use and no requirement for specialized equipment for its operation [14], [15], [16]. However, the device fails to satisfy the needs of biomedical researchers to efficiently modulate oxygen tensions rapidly and over multiple conditions as a separate chamber is required per condition. Several groups have developed devices for controlling the oxygen tension experienced by cell cultures that are aimed to improve upon the hypoxic chamber [3], [17], [18], [19], [20], [21]. Despite advancements in system equilibration time and the ability to establish oxygen gradients, many of these devices require very specific parameters for operation, including the need for complex fluid handling and often times electric controls [20]. The requirement of specialized knowledge and equipment for their use is unattractive to many labs.

**Figure 1:**
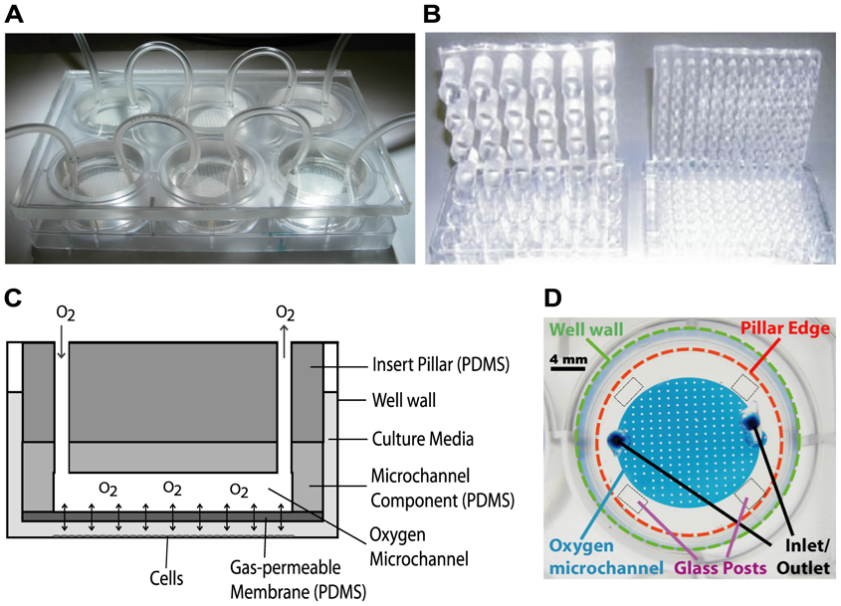
Schematic and diagrams illustrating device features. The oxygen insert device is fabricated by conventional photolithography (microfluidic network), replica molding (microfluidic network and insert scaffold), and defined spinning of PDMS (gas-permeable membrane). A) The oxygen device nested into a 6-well plate. B) Examples of 24 and 96-well pillar arrays. C) A cross-sectional schematic of a pillar. Oxygen flows into the device through the inlet and travels across a microfluidic network at the bottom of the pillar. Oxygen can freely diffuse across the gas-permeable PDMS membrane at the bottom of the pillar and dissolve into the culture media. D) A macroscope image showing the various features of a single-channel pillar from above, with bonded glass posts for the equilibration studies.

**Figure 2:**
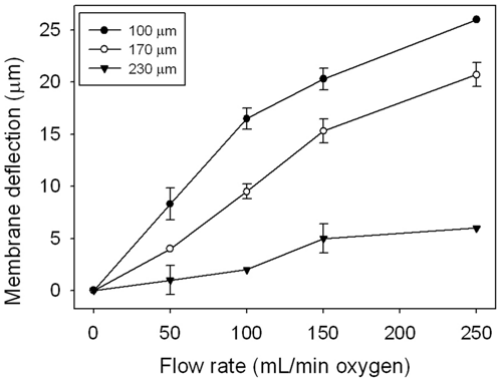
Deflection of PDMS membranes of varying thicknesses. Deflection distance was inversely proportional to membrane thickness, with the 100 µm membrane deflecting the most and the 230 µm membrane deflecting the least. The data indicates that deflection of the 100 µm is negligible for operational flow rates (∼25 mL/min) relative to the overall gap distance between the membrane and cells.

A new device with more precise temporal and spatial control over gas concentrations experienced by the cell, while possessing a smaller lab footprint than many other current cell oxygenation devices, could be very beneficial to the scientific community. To meet this demand a new device has been fabricated consisting of a polydimethylsiloxane (PDMS) insert that nests into a standard multiwell plate as shown in figure 1a. The insert contains a series of pillars matching the number and spacing of wells of the plate it is designed to nest into. A 6-well version was used for proof of concept purposes; however, the strategy can be easily expanded to higher density multiwell plates (e.g. 12, 24, 48 or 96-wells) as shown in figure 1b. Oxygen is injected through microchannels embedded at the base of each pillar and is separated from the fluidic contents of the culture well by a 100 µm gas-permeable PDMS membrane, as shown in a schematic cross-section of the device in figure 1c. The bottom of the membrane can be fixed at various distances from the substrate of the well by employing spacers or altering the length of the pillar. The oxygen microchannels connect to conventional gas cylinders which provide the pressure to deliver the gas throughout the insert. The insert acts as a sink or source of oxygen depending on the concentration of the oxygen in the microchannels, and is immersed in the cell culture medium of the well. The gas is delivered to the cell monolayer through simple diffusion of oxygen across the PDMS membrane and dissolves into the culture media of the multiwell plate. The cultured cells are never in contact with gas and hence issues with gas bubbles disrupting the integrity of the cell membrane are avoided. The oxygenation routing and pillar microchannels are fabricated through standard soft lithographic techniques [22] and the pillar array is fabricated through casting PDMS into a machined Delran mold. Figure 1c also depicts the cell monolayer at the bottom of the well positioned approximately 200 µm from the bottom of a pillar. This close spacing between the PDMS membrane and cell monolayer allows for rapid diffusion to impose steady state gradients of oxygen across the well within minutes and is not altered by the slight deflection of the elastomeric flexible membrane as indicated by figure 2. Many oxygenation profiles can be generated depending on the layout of the microfluidic oxygenation channels at the base of the pillar.

PDMS is used as the material of the insert due to its optical clarity, gas permeability (D_oxygen in PDMS_ = 3×10^−5^ cm^2^/s), and ease of rapid prototyping. However, several recent studies have highlighted the ability of PDMS to absorb water and hydrophobic soluble factors [23], [24], [25], [26]. Only the membrane component of our platform needs to be made with PDMS due to its excellent oxygen permeability and the bulk of our platform (including the gas microchannels and pillar array) can be fabricated with alternative polymers more resistant to hydration and absorption such as polymethylmethacrylate, cyclic olefin copolymer, or polycarbonate. Our initial studies rely on PDMS due to the ease of rapid prototyping and have taken measures to avoid hydration of the manifold from altering the experimental conditions. Water loss through absorption into PDMS would be detrimental in cell culture that relies on specific concentrations of solutes to maintain pH and nutrient levels. Specifically the device is pre-hydrated through immersion in cell media or water prior to experimentation. While PDMS is not an ideal material for cellular studies, these measures can be taken to improve its functionality.

## Results

### PDMS membrane deflection is negligible

Due to the small gap size between the device and cells, the deflection of the PDMS membrane due to gas infusion pressure needed to be assessed, as shown in figure 2. The device was laid on its side and deflection distance was measured using microscopy. As expected, deflection distance was inversely proportional to PDMS membrane thickness and directly proportional to oxygen flow rate. This data indicated that the PDMS membrane deflection is negligible as the device's operational flow rates (80 mL/min for initial equilibration and 25 mL/min for concentration maintenance) do not deflect the membrane enough for contact with the underlying cells. Even at the highest flow rate (250 mL/min) and 100 µm thickness, the membrane would still only deflect less than 15% of the distance to the cells.

### Device oxygen concentration validation

**Figure 3:**
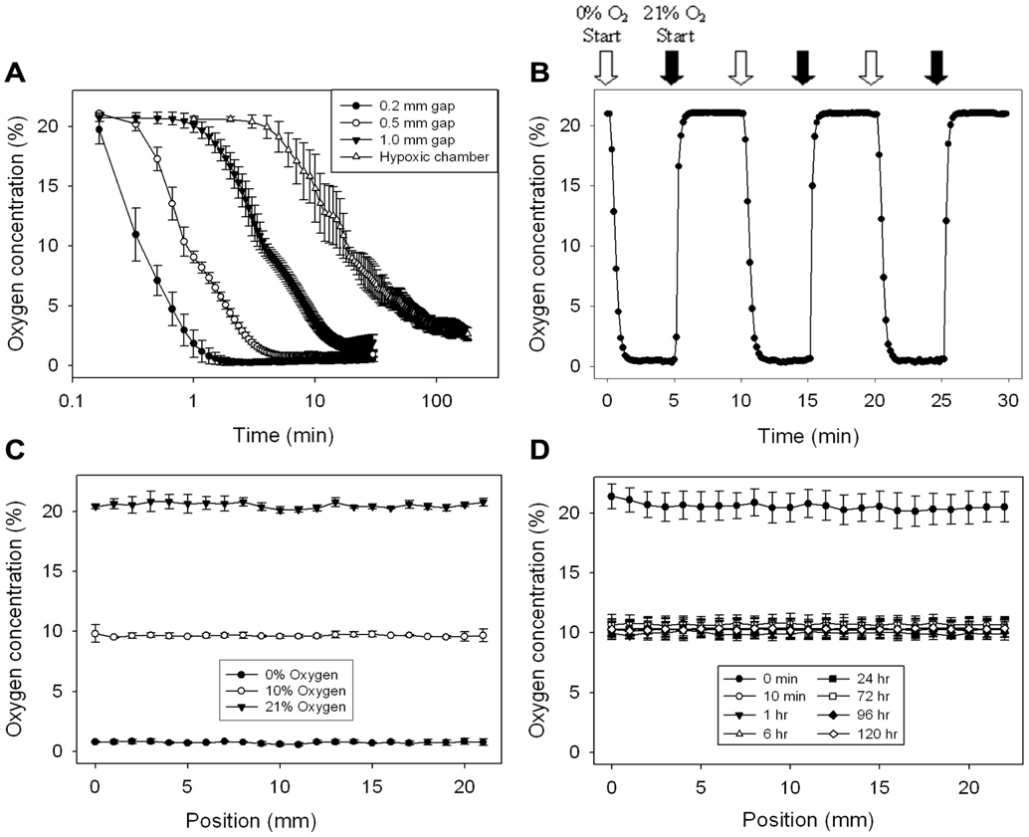
Validation of the device with oxygen sensors. Oxygen tension within each well was characterized using a planar ruthenium oxygen sensor. All oxygen mixtures contained balanced nitrogen and 5% CO_2_ for media buffering. A) Plot illustrating the effect of post height, and thus oxygen diffusion distance between the membrane and cells, on the equilibration time and effectiveness. Heights were established by cut-glass posts bound to the bottom of the device. All three post sizes yield equilibration times much improved over the hypoxic chamber. Note that time is on a log scale. B) Plot depicting the rapid oxygen equilibration response time of the 0.2 mm gap device. C) Multi-position linescans were also taken across the well under the microchannel to ensure homogeneity of the oxygen concentration introduced by the device. Graph depicts the oxygen concentration measured after infusing 0%, 10%, and 21% oxygen for 10 min. D) Device effectively maintains 10% oxygen over 5 days.

A planar ruthenium fluorescence oxygen sensor (FOXY slide) was used for quantifying the oxygen concentration at the well substrate. The fluorescence of the ruthenium complex is quenched in the presence of oxygen, thus oxygen concentration can be calculated via measurement of fluorescent intensity. The 0% oxygen equilibration experiments demonstrated that the device can rapidly change the oxygen concentration at the well substrate as shown in figure 3a. For a membrane-well bottom spacing of 0.2 mm, the device equilibrated to less than 0.5% oxygen in about 1.5 min. Equilibration took slightly longer and was less complete for the 0.5 mm spacing, taking about 5 min to achieve roughly 1% oxygen. For the 1 mm spacing, the device equilibrated to approximately 2% oxygen in about 15 min. Equilibration for the hypoxic chamber was orders of magnitude slower, taking about 3 h to reach approximately 2.5% oxygen which is in agreement with previously published data [27]. Figure 3b depicts the rapid response of the oxygen concentration at the bottom of the well when switching between two oxygenation conditions, which is not possible with the standard hypoxic chamber due to long equilibration times.

Intensity measurements were also taken across the width of the channel at specific positions to ensure homogeneity of oxygen concentrations across the well. For the single channel device, infused 0%, 10%, and 21% oxygen were measured at 20 min as shown in figure 3c. The oxygen concentration maintained good homogeneity with no considerable increase near the microchannel boundary. The device was also tested for maintenance of 10% oxygen over 5 days as shown in figure 3d. After the initial baseline point, all measurement time points yielded 10% oxygen without prolonged immersion in media altering the delivered oxygen.

**Figure 4:**
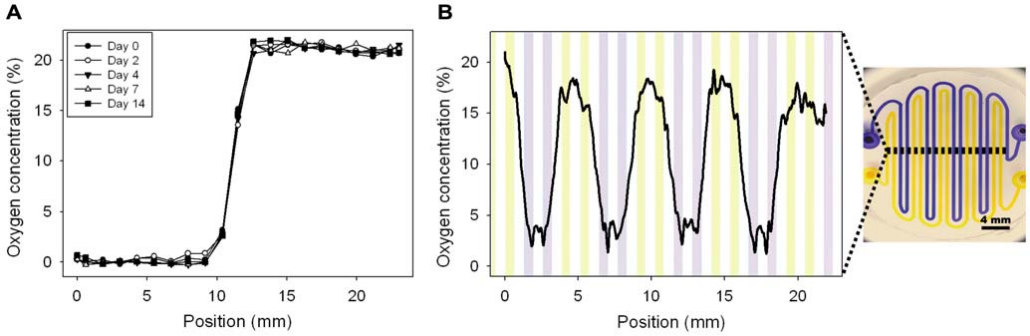
Experimentation with more complex oxygen microchannel designs. A) Dual-condition microchannel setup yields a stable 0% and 21% oxygen profile over 14 days. B) An interdigitated and winding pattern of 500 µm width microchannels extending across the pillar results in a cyclic oxygen profile. Note that the data only depicts one representative trial as microchannel alignment was difficult.

Devices were also fabricated with more complex microchannel designs, which provided enhanced spatial control over the oxygenation across the well. A dual-microchannel device effectively maintained both 0% and 21% oxygen across a single well for 14 days as shown in figure 4a, again demonstrating that media diffusion into the pillar did not adversely affect the oxygen concentration.

To push the limits of this approach, a cyclic oxygenation profile was demonstrated across a single well as shown in figure 4b. While the device was unable to generate the minimum and maximum conditions as close as the single condition device as shown in figure 3, the device did successfully generate distinct oxygenation zones with a gradient of oxygen tension separating each zone. The diffusion of gas from one microchannel to the next was minimized by maintaining the gas flow rate much higher than the oxygen diffusion rate. These initial demonstrations can be used as a starting point for many other complex oxygen profiles to expand the number of experimental possibilities for the biomedical researcher.

### Common sterilization methods do not alter device gas delivery

**Figure 5:**
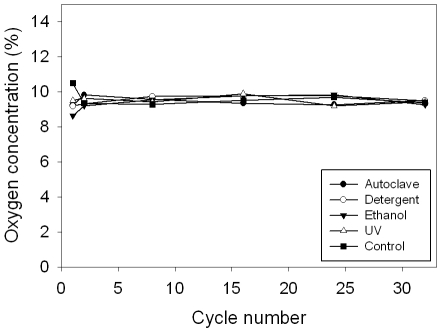
Common sterilization methods do not alter device's ability to deliver oxygen. An important aspect of any cell culture tool is the ability to sterilize it without the loss of function. Four separate devices were exposed to each of the commonly available sterilization methods over 32 cycles, with an untreated control. The oxygen concentration at the bottom of the well was measured with the fluorescent oxygen probe with infused 10% O_2_ on cycle 1, 2, 8, 16, 24, and 32. No sterilization method had a significant effect on device function compared to control.

Since the device will be used for cell culture experiments, sterilization is required before its application. Four sterilization methods were used to assess the affect of sterilization on diffusion of oxygen through the membrane. No significant difference was observed between any of the sterilization methods and the control condition after 32 cycles, demonstrating that the device can be sterilized without altering its function as shown in figure 5.

### Cellular proliferation and transcription factors are modulated with the device

**Figure 6:**
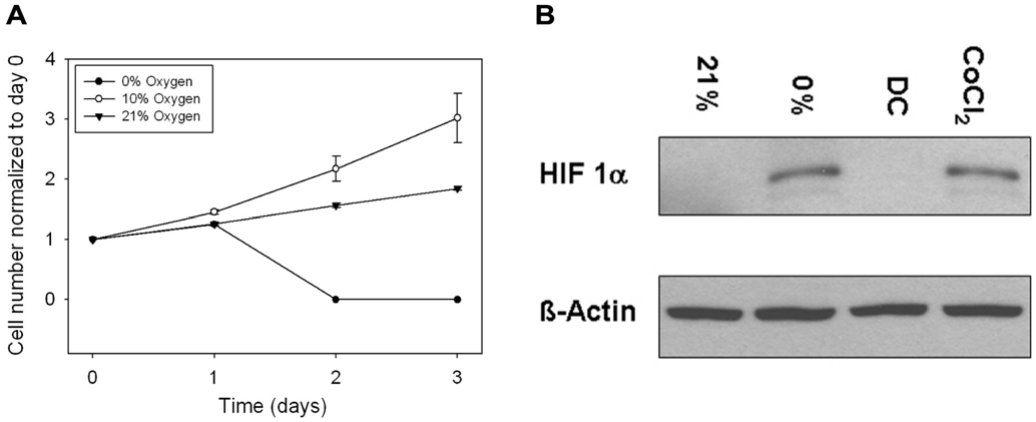
Validation of the device with cellular models. A) Human adult dermal fibroblast cells were used as a cellular model to study proliferation in response to oxygen concentrations established by our device. Cells were seeded in the wells of a 6-well plate and cultured to confluency. The device was removed from the plate at 24, 48, 72, and 96 h to briefly exchange the culture media and to take images of the cells for later cell counting. The plot depicts the proliferation of cells. Data is presented as cell number normalized to baseline (day 0) over the course of 4 days for cells continually exposed to 0%, 10%, and 21% O2. Proliferation was highest in 10% O_2_ (as expected). No cells survived after 4 days in 0% infused oxygen. B) Human osteosarcoma cells (U2OS) were seeded in a 6-well plate to ∼85% confluency and exposed to hypoxia and normoxia for 24 h using the hypoxic insert device. Control cell media was supplemented with 10 µM CoCl_2_, which mimics the hypoxic state and results in HIF-1α upregulation [27]. Cells cultured in atmospheric oxygen without exposure to the device served as the device control (DC). Western analysis was used to qualify regulation of HIF-1α expression. The figure depicts upregulation of HIF-1α in cells exposed to hypoxia using the device, relative to the no device control and 21% oxygen. The device 0% oxygen band intensity is comparable to the CoCl_2_ control.

The device's ability to modulate cellular behavior including proliferation and HIF-1α expression was also investigated to validate the device's compatibility with standard cell culture techniques. The device successfully regulated human dermal fibroblast proliferation as shown in figure 6a. As expected, proliferation was highest in 10% oxygen [28], [29]. No cells survived after 4 days in 0% infused oxygen.

For qualifying HIF-1α expression in human osteosarcoma cells (U2OS) using Western analysis, cell lysate was immediately extracted after removal of the hypoxic insert device. Lysate protein was separated on an SDS-PAGE gel and transferred to a membrane for immunoblotting using the H1α67 primary antibody. Western analysis confirmed that HIF-1α was upregulated in cells exposed to hypoxia as compared to the 21% oxygen control cells as shown in figure 6b. These experiments using cellular models affirm that the device functions appropriately for *in vitro* cell experiments.

## Discussion

As the data demonstrate, the hypoxic insert device is effective at both controlling the temporal and spatial concentration of oxygen in a 6-well plate. Fast equilibration time and complex oxygen concentration patterning based on the microchannel design expands the experimental possibilities to include studies not possible with conventional technologies. In this study, several oxygen concentration profiles were achieved and many more are possible, including complex gradients and shifting oxygen regimes. As mentioned previously, the gold standard for hypoxia exposure is the hypoxic chamber, which takes hours to equilibrate, cannot achieve extremely hypoxic regimes (<2%), and only permits a single oxygen concentration within the same cell population. However, the device presented here reduces the equilibration time by orders of magnitude, yields concentrations below 0.5% oxygen, and permits very complex oxygen profiles across the same well. It is important to note these experiments did not contain cells, which would further deplete the available oxygen. Future improvements of the device are planned, including on-device custom gas mixing, media perfusion ports for slow exchange of cell media, and secondary gas ports for areas surrounding the pillar. Finally, the current device iteration was used with 6-well plates, but our lab is designing devices for use in 12, 24, and even 96-well plates to increase experimental throughput, in addition to Petri dishes and larger scale culture vessels.

In addition to general *in vitro* studies involving control over gas concentration, the hypoxia insert device is particularly advantageous for the enormous gamut of applications requiring transient effects or complex oxygen profiles. Ischemia reperfusion injury, as in the case of cardiac (myocardial infarction) or cerebral (stroke) tissue, is one such research application of the device due to its highly effective temporal control over oxygen concentration, permitting more accurate investigation of the causes for tissue necrosis post-reperfusion. Other topics benefiting from the more rapid equilibration response of the device include sleep apnea and cancer cell research. In fact, hypoxia and activation of the HIF-1α pathway are characteristic of many types of tumors [30] and can be indicative of tumor aggressiveness [31]. More recently, intermittent hypoxia has been shown to induce elevated levels of HIF-1α transcription [32] and tumor invasiness [13] over continuous hypoxia. Thus cyclic reoxygenation plays a role in cellular response to hypoxia and faster system oxygen concentration response time would expand the experimental possibilities. As mentioned earlier, fields of research benefiting from the ability to generate complex oxygen gradients include but are certainly not limited to liver zonation, stem cell niche, drug toxicity, and developmental biology. The number of experiments that will benefit from enhanced spatial and temporal control over oxygen concentration offered by the hypoxia device is substantial and will likely extend to new areas of research that were either too difficult to conduct with the current technologies or were simply not possible.

## Materials and Methods

### Device fabrication

The oxygen insert device was fabricated using common soft lithography techniques in a 3-step process: 1) gas-permeable membrane, 2) the microfluidic network, and 3) the insert scaffold as shown in figure S1a. The overall pillar length was tailored to achieve a desired height separation between the bottom of the membrane and the seeded cells, depending on the experiment.

The 100 µm-thick gas-permeable PDMS membrane was made by spinning premixed 10∶1 ratio of PDMS prepolymer and curing agent (Sylgard 184 kit, Dow Corning) on a thoroughly cleaned silicon wafer vacuum-adhered to a precision spinner (Laurel). The dependence of PDMS thickness on spin speed and duration is depicted in figure S1b. The PDMS-coated wafer was spun at 500 RPM for 10 seconds to initially spread the PDMS droplet, and then 900 RPM for 30 seconds to attain the desired thickness. The PDMS-coated wafer was then placed on top of a leveled hot plate set to 75°C and heated for two hours to cure the PDMS. After curing, a small circle of PDMS is removed from the wafer-adhered sheet and placed on top of a transparency film to facilitate handling when bonding to the device.

The microfluidic network was designed in AutoCAD and printed onto high resolution (5080 dpi) transparencies (Fineline Imaging). The transparency is used as a photomask to selectively crosslink a photoresist prespun to a desired thickness on a silicon wafer. Briefly, SU-8 2150 photoresist (Microchem) was spun to achieve a thickness of 200 µm in accordance to the manufacturer's protocol. This resist was then selectively exposed to UV light through the photomask to selectively polymerize the SU-8. Finally, uncross-linked regions of the photoresist were removed by washing the wafer in SU-8 developer solution. Once the SU-8 negative mold master was fabricated, PDMS was poured onto the master to generate a positive mold at the desired thickness. The PDMS mixture was cured for 2 hours at 75°C. Once cured, a section the size of the device pillar was removed from the molded PDMS.

The actual insert scaffold component of the device was fabricated by molding in a machined polycarbonate scaffold as shown in figure S1c. The PDMS was cured overnight in a convection oven set to 75°C.

After fabrication of the three components was completed, the device itself was ready to be assembled. First, the insert component and the microfluidic network component were bonded after surface exposure to oxygen plasma and 1 h incubation. For the 6-well plate device, 6 microfluidic network components were bonded to the 6 pillars of the insert. Then, a cork-borer was used to puncture holes connecting the top of the insert component to the bottom of the microfluidic network component. These vias allowed introduction of gases into the microfluidic channels of the device. Then, the insert/microfluidic network component and membrane component were bonded after exposure to oxygen plasma and incubated for 1 h. For some of the oxygen validation studies, cut glass posts from coverslips or microscope slides were bonded to the membrane under the periphery of the microchannel to alter the spacing between the substrate of the pillar and the substrate of the well. Note that glass posts were utilized only for the validation studies so that the same pillar mold (and thus length) could be used for a variety of gap sizes. The pillar length determines the media diffusion distance in the functional version of the device.

### PDMS membrane deflection

Due to the small gap size between the device and cells, the deflection of the PDMS membrane due to gas infusion pressure needed to be assessed. Three different devices were made to examine the effect of PDMS membrane thickness on deflection. Each membrane thickness was examined with four different infused oxygen flow rates. The device was laid on its side and deflection distance was measured using microscopy (see microscope system information in the following oxygen concentration validation section).

### Oxygen concentration validation in the device

FOXY slides (FOXY-SGS, Ocean Optics) were used for quantifying the oxygen concentration at the well substrate [33]. Recall that the oxygen concentration is inversely proportional to the fluorescent intensity of the slide. Briefly, the slide was placed in the wells of a 6-well plate, simulating where the cells would be present. Oxygen concentrations of 0%, 10%, and 21% (5% CO_2_, balanced nitrogen tanks) were introduced into the channels of the device and oxygen concentration was measured via fluorescence. Note that 5% CO_2_ is needed for culture media pH buffering. However, cells were not used in these validation studies in order to test the functionality of the device alone, as metabolic oxygen uptake can vary dramatically between cell types and density.

A fluorescence-equipped Olympus IX71 microscope, charged-coupled device camera (QImaging Retiga-SRV) and SlideBook image acquisition software (v4.2.0.9) were used for capturing images. All images were acquired at room temperature using a FOXY-compatible fluorescent filter (Olympus 31020) with an excitation wavelength of 475 nm and emission wavelength of 600 nm. Images were taken at 10X (UPlanSApo 10x/0.40) for all experiments except for the cyclic profile device, where images were captured at 2X (PlanApo 2x/0.08). A motorized stage (Prior ProScan II) with x, y, and z control was used to store and return to previous image capture locations. A 75W Xenon burner (Olympus U-LH75X) was used to illuminate the FOXY slides. Image intensities were exported using SlideBook for later analysis. DI water was used as the diffusion medium for FOXY experiments.

To establish a calibration curve at each slide position, a device without the membrane was placed directly against the FOXY slide so that the bottom of the microchannel was the fluorescence oxygen sensor itself. Thus, the slide was directly exposed to the introduced oxygen without the media and membrane in the diffusion path. Two linear curve equations were fitted to the three data points using Microsoft Excel; one from 0% to 10% percent oxygen, and the other from 10% to 21% oxygen. This method proved to be more accurate than using a second degree polynomial curve fit as described by the manufacturer, which tended to underestimate lower oxygen concentrations.

For the 0% oxygen equilibration studies, slide-membrane separations of 0.2 mm, 0.5 mm, and 1.0 mm were used to assess the effect of oxygen diffusion distance on measured oxygen concentration at the well surface. The height separation was precisely controlled by bonding cut-glass posts to the bottom of the pillar. The overall pillar length was made longer to ensure that the post height was actually determining the diffusion distance and the pillar was not merely suspended from the top of the well. 0% oxygen was introduced into the channel and fluorescence intensity measurements were taken every 10 seconds for 30 min. A hypoxic chamber was also used as a comparison to demonstrate the effectiveness of the device. Briefly, the hypoxic chamber was rapidly flushed with 0% oxygen and then a steady influx of 0% oxygen was maintained for 3 h. Intensity measurements were taken every minute and reflected the average intensity value of the entire image

Multi-position linescans were also taken across the well under the microchannel to ensure homogeneity of the oxygen concentration. Infused oxygen concentrations of 0%, 10%, and 21% were measured after 20 min in the device. The ability to maintain 10% oxygen over 5 days was also examined using the same setup as for the other linescans.

One important feature of the device is the ability to precisely control the spatial oxygen concentration across the well by changing the design of the microchannel at the bottom of the pillar. A dual-channel device was fabricated to validate the effect of two concentrations across a single well. Oxygen concentrations of 0% and 21% oxygen were introduced into the channels of the device and oxygen concentration was measured via fluorescence across the well at fixed positions similarly to the above experiments. The experiment was run for 14 days to evaluate the effect of media diffusion into the device on oxygen concentration.

A cyclic profile device was also fabricated to validate the ability of the device to generate complex profiles across a well. Two interdigitated and winding microchannels of 500 µm width extended across the pillar to generate a cyclic oxygen profile using two of the three oxygen concentrations available at a time. Four images at 2X that spanned the entire well were captured, linescans were taken across each image, and the exported intensity data was stitched together. Note that the linescan reflects the inhomogeneity of the ruthenium embedded in the FOXY slide more than previous quantifications which averaged over a larger area for a single value.

### Effect of common sterilization methods on the device

An important aspect of any cell culture tool is the ability to sterilize it without the loss of function. Four sterilization methods were used to assess the affect of sterilization on diffusion of oxygen through the membrane: 1) Autoclave at 121°C for 15 min, 2) Soak in Dri-Clean detergent at 52°C for 15 min, 3) Soak in 70% ethanol for 15 min, 4) Exposure to culture hood ultraviolet sterilization light at 253.7 nm for 2 h. Four separate devices were exposed to each of the sterilization methods over 32 cycles. The oxygen concentration at the bottom of the well was measured with the fluorescent oxygen probe at 10% oxygen on cycle 1, 2, 8, 16, 24, and 32.

### Effect of oxygen concentration on fibroblast proliferation

Human adult dermal fibroblast cells (Sciencell) were used as a cellular model to study proliferation in response to oxygen concentrations established by our device in a 100 µm diffusion gap configuration. Cells were expanded in fibroblast culture media supplemented with 2% Fetal Bovine Serum, 1% Penicillin/Streptomycin, and 1% Fibroblast Growth Serum (Sciencell). Fibroblasts were seeded into the wells of a 6-well plate at a density of 2.5×10^3^ cells/cm^2^. Media was then changed the next day immediately prior to introduction of 0%, 10%, and 21% oxygen into the device. Experiments were conducted in a humidified incubator (95% relative humidity, 37°C) to prevent media evaporation and maintain proper physiological temperature. The insert was removed from the plate at 24, 48, 72, and 96 h to briefly exchange the culture media and to take images of the cells for later cell counting. Once the media was changed and five brightfield microscopy images per well were taken, the cell-seeded plate was placed back into the culture incubator and the experiment was continued. Cells were later counted using the acquired images.

### Western analysis of HIF-1a

In order to test the physiological relevance of the hypoxic setup, U2OS cells were plated at 80% confluency in DMEM media supplemented with 10% Fetal Bovine Serum and Penicillin/Streptomycin. The cell-seeded plate was placed in the incubator and the device was nested and exposed to either 21% or <0.5% oxygen. After 24 hours, cells were harvested immediately in lysis buffer (1 mM EDTA, 0.15 M NaCl, 0.05 M Tris-Hcl, 0.5% Triton-X) and placed in dry ice in order to preserve protein integrity. Fifty µg of protein lysate was separated on an 8% SDS-PAGE gel and HIF-1α was detected using the mouse monoclonal H1alpha67 antibody (Abcam, Cambridge Massachusetts, 1∶1000). U2OS cells treated in 10 µM CoCl_2_ for 24 hours serves as a control as CoCl_2_ has been previously shown to mimic the hypoxic state and as a result cause induction of HIF-1 µ [27].

### Statistical analysis

All experiments were conducted at least three times for proper statistical analysis unless otherwise noted in the text. Graphs depict the average value with the error bars indicating standard deviation.

## Supporting Information

Figure S1 Fabrication of the device. Polydimethylsiloxane (PDMS) is used for the device due to its biocompatible, moldability, and most importantly, gas-permeability. A) Overall schematic of the fabrication process. The oxygen microfluidic channels are fabricated using standard SU-8 photolithography. PDMS is then replica molded from the negative SU-8 master to create the microfluidic network. For the oxygen validation studies, cut glass posts were bonded to the bottom of the device outside of the microchannel area for precise establishment of diffusion distance. B) The gas-permeable membrane is made by precision spinning PDMS prepolymer mix to 100 Âµm. C) The pillar array is casted in a polycarbonate mold.
(1.00 MB DOC)
