# Supplementary figures and images for: Modulating Temporal and Spatial Oxygenation over Adherent Cellular Cultures
Source: PLoS One. 2009 Sep 3;4(9):e6891. doi: 10.1371/journal.pone.0006891 (PMC2731542; doi:10.1371/journal.pone.0006891)

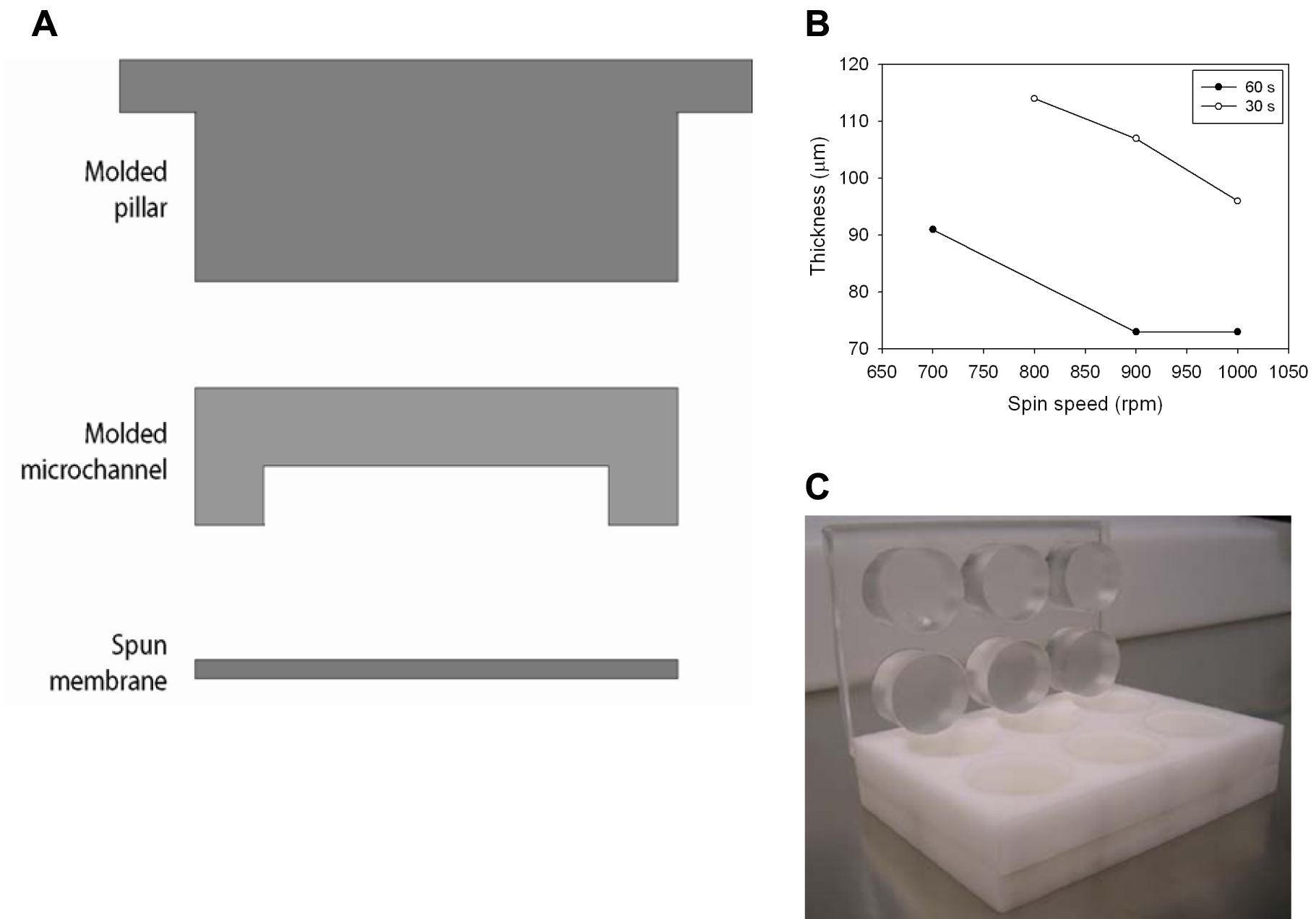

Supplement: Figure S1 — Fabrication of the device. Polydimethylsiloxane (PDMS) is used for the device due to its biocompatible, moldability, and most importantly, gas-permeability. A) Overall schematic of the fabrication process. The oxygen microfluidic channels are fabricated using standard SU-8 photolithography. PDMS is then replica molded from the negative SU-8 master to create the microfluidic network. For the oxygen validation studies, cut glass posts were bonded to the bottom of the device outside of the microchannel area for precise establishment of diffusion distance. B) The gas-permeable membrane is made by precision spinning PDMS prepolymer mix to 100 Âµm. C) The pillar array is casted in a polycarbonate mold. (1.00 MB DOC) [file pone.0006891.s001.tif]
